# The Two Different Isoforms of the RSC Chromatin Remodeling Complex Play Distinct Roles in DNA Damage Responses

**DOI:** 10.1371/journal.pone.0032016

**Published:** 2012-02-16

**Authors:** Anna L. Chambers, Peter M. Brownlee, Samuel C. Durley, Tracey Beacham, Nicholas A. Kent, Jessica A. Downs

**Affiliations:** 1 Medical Research Council Genome Damage and Stability Centre, University of Sussex, Falmer, Brighton, United Kingdom; 2 Cardiff School of Biosciences, Cardiff University, Cardiff, United Kingdom; University of Minnesota, United States of America

## Abstract

The RSC chromatin remodeling complex has been implicated in contributing to DNA double-strand break (DSB) repair in a number of studies. Both survival and levels of H2A phosphorylation in response to damage are reduced in the absence of RSC. Importantly, there is evidence for two isoforms of this complex, defined by the presence of either Rsc1 or Rsc2. Here, we investigated whether the two isoforms of RSC provide distinct contributions to DNA damage responses. First, we established that the two isoforms of RSC differ in the presence of Rsc1 or Rsc2 but otherwise have the same subunit composition. We found that both *rsc1* and *rsc2* mutant strains have intact DNA damage-induced checkpoint activity and transcriptional induction. In addition, both strains show reduced non-homologous end joining activity and have a similar spectrum of DSB repair junctions, suggesting perhaps that the two complexes provide the same functions. However, the hypersensitivity of a *rsc1* strain cannot be complemented with an extra copy of *RSC2*, and likewise, the hypersensitivity of the *rsc2* strain remains unchanged when an additional copy of *RSC1* is present, indicating that the two proteins are unable to functionally compensate for one another in DNA damage responses. Rsc1, but not Rsc2, is required for nucleosome sliding flanking a DNA DSB. Interestingly, while swapping the domains from Rsc1 into the Rsc2 protein does not compromise hypersensitivity to DNA damage suggesting they are functionally interchangeable, the BAH domain from Rsc1 confers upon Rsc2 the ability to remodel chromatin at a DNA break. These data demonstrate that, despite the similarity between Rsc1 and Rsc2, the two different isoforms of RSC provide distinct functions in DNA damage responses, and that at least part of the functional specificity is dictated by the BAH domains.

## Introduction

Access to chromatin can be regulated by covalent post-translational modification of histone proteins and by the action of ATP-dependent remodeling complexes. ATP-dependent remodelers are large multi-subunit complexes that couple ATP hydrolysis to movement of histones or nucleosomes. A number of different remodeling activities can be performed by these complexes, including exchange or incorporation of core histones or histone variants, eviction of histones or nucleosomes and repositioning or sliding of nucleosomes [1].

Chromatin remodeling activities are known to facilitate the repair of DNA double-strand breaks (DSBs). One such activity is the RSC (Remodels the Structure of Chromatin) complex originally identified by Cairns *et al* [2]. The catalytic activity is provided by Sth1, which is encoded by an essential gene, and there are currently 17 known subunits (Sth1, Rsc1, Rsc2, Rsc3, Rsc4, Rsc6, Rsc7/Npl6, Rsc8, Rsc9, Sfh1, Arp7, Arp9, Rsc30, Htl1, Rtt102, Rsc58 and Ldb7).

There appear to be at least two distinct isoforms of the complex, containing either Rsc1 or Rsc2 [3]. *RSC1* and *RSC2* encode proteins with highly similar domain organization and 62% amino acid similarity [3]. Both proteins have two bromodomains separated by an AT hook followed by a bromo-adjacent homology (BAH) domain. While Rsc1 and Rsc2 complexes appear to occupy identical regions of the genome when assayed by ChIP on chip [4], *rsc1* and *rsc2* mutant strains have overlapping but not identical cellular phenotypes [3], [5], [6], [7] suggesting that they do not act entirely redundantly. This may be due to transient and dynamic differences in Rsc1 and Rsc2 binding or a difference in dosage since Rsc2 is estimated to be ten-fold more abundant than Rsc1 [3]. However, a *rsc1*/*rsc2* double mutant is lethal, suggesting that there is at least some functional overlap.

Both Rsc1 and Rsc2 have been implicated in DNA DSB repair. Strains lacking either *RSC1* or *RSC2* are hypersensitive to a variety of DNA damaging agents [8], [9], [10], and both *rsc1* and *rsc2* mutant strains were reported to have problems with homologous recombination (HR) [9]. While *rsc1* and *rsc2* mutant strains were not directly tested in non-homologous end joining (NHEJ) assays, strains lacking subunits common to both isoforms were found to have defective NHEJ [10], indicating that at least one of the two isoforms is important for this pathway. The catalytic subunit of RSC accumulates at DNA DSBs by ChIP [9], [10], suggesting that at least one of the two isoforms is recruited to breaks.

Once recruited to a DNA DSB created at the MAT locus, we and others find that the chromatin flanking the break is remodeled in a RSC-dependent manner [8], [11]. We found that the remodeling event was dependent on Rsc1 but not Rsc2 [8]. Using a different assay, however, Lee and colleagues found the remodeling was dependent on Rsc2 (Rsc1 was not tested) [11]. These data suggest that perhaps both isoforms remodel nucleosomes flanking the DNA DSB, but it is not clear whether they function interchangeably.

Here, we present evidence that Rsc1 and Rsc2 are providing distinct functions to DNA DSB repair. Both isoforms contribute to repair by NHEJ, and have a similar spectrum of repair junctions, which is substantially different from wt. In addition, the domains from Rsc1 can largely function in place of the equivalent Rsc2 domains in DNA repair assays. However, increasing the dosage of either gene cannot compensate for loss of the other. Moreover, we extended our nucleosome remodeling analysis to a second DSB site in the genome, and again find that only *rsc1* mutant strains show a defect in this assay, suggesting that the two proteins provide distinct functions. In support of this, when the BAH domain from Rsc1 is swapped into a Rsc2-expressing construct, the Rsc2^BAH^ chimeric protein is able to remodel nucleosomes flanking a DNA DSB, while still retaining Rsc2-specific remodeling activity prior to DSB formation at the MAT locus. Together, these data demonstrate that Rsc1 and Rsc2 have distinct as well as overlapping functions in DNA damage responses, and that the BAH domains are important for determining the functional specificity.

## Results

### Rsc1 and Rsc2 are present in two separate isoforms of RSC

**Figure 1:**
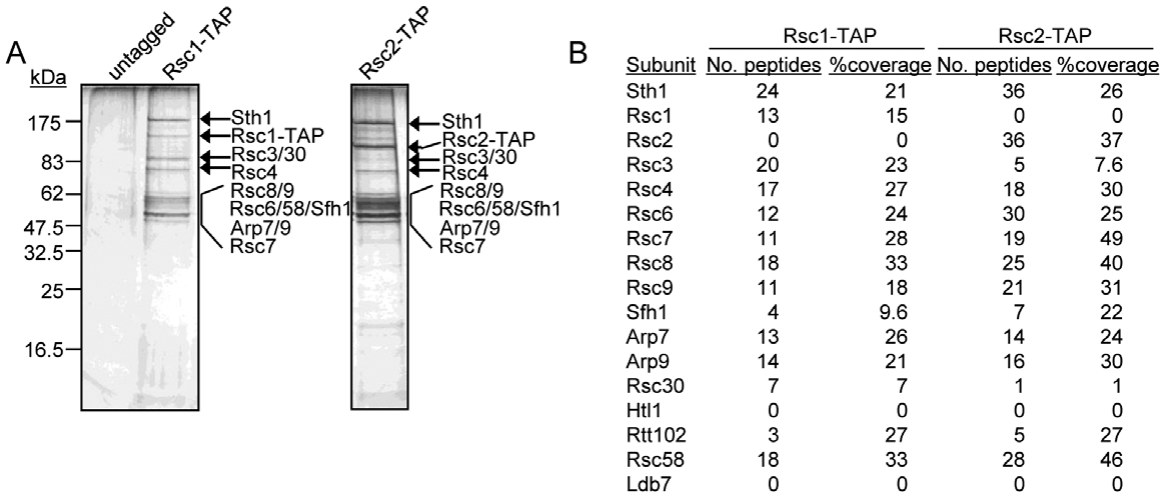
Rsc1 and Rsc2 define two distinct RSC isoforms. (A) Tandem affinity purification from a strain with TAP-tagged Rsc1, TAP-tagged Rsc2, or from an untagged strain. (B) Results from mass spectroscopy analysis of the purified complexes from A.

A previous study indicated that there are two separate isoforms of RSC containing either Rsc1 or Rsc2, although the subunit composition of each isoform was not determined [3]. To investigate whether the two isoforms of RSC are otherwise identical, we made use of strains containing a C-terminal tandem affinity purification (TAP) tag on either Rsc1 or Rsc2. Affinity purifications were performed from cell extracts prepared from strains expressing Rsc1-TAP, Rsc2-TAP, or from an untagged wild-type control strain (Figure 1A). The purified complexes were analyzed by mass spectroscopy and the results are presented in Figure 1B. When we used TAP tagged Rsc1, we did not identify Rsc2, and conversely, when we used TAP tagged Rsc2, we did not detect Rsc1 present in the purified complexes. These data indicate that the vast majority of Rsc1 and Rsc2 are in two distinct isoforms of RSC, consistent with previous work [3]. In addition, we identified all other subunits of RSC in both purifications with the exception of Htl1 and Ldb7, which are among the smallest subunits of RSC (9.1 and 19.7 kDa, respectively). This suggests that the two complexes are identical with the exception of the presence of Rsc1 or Rsc2. However, it has been reported that Rsc3 and Rsc30, which form a heterodimer, preferentially associate with Rsc1 [12]. Consistent with this possibility, there were fewer peptides from Rsc3 and Rsc30 detected in the Rsc2 purification relative to the Rsc1 purification (Figure 1B), although Rsc3 and Rsc30 are clearly present in both.

### Neither Rsc1 nor Rsc2 is required for checkpoint activation

**Figure 2:**
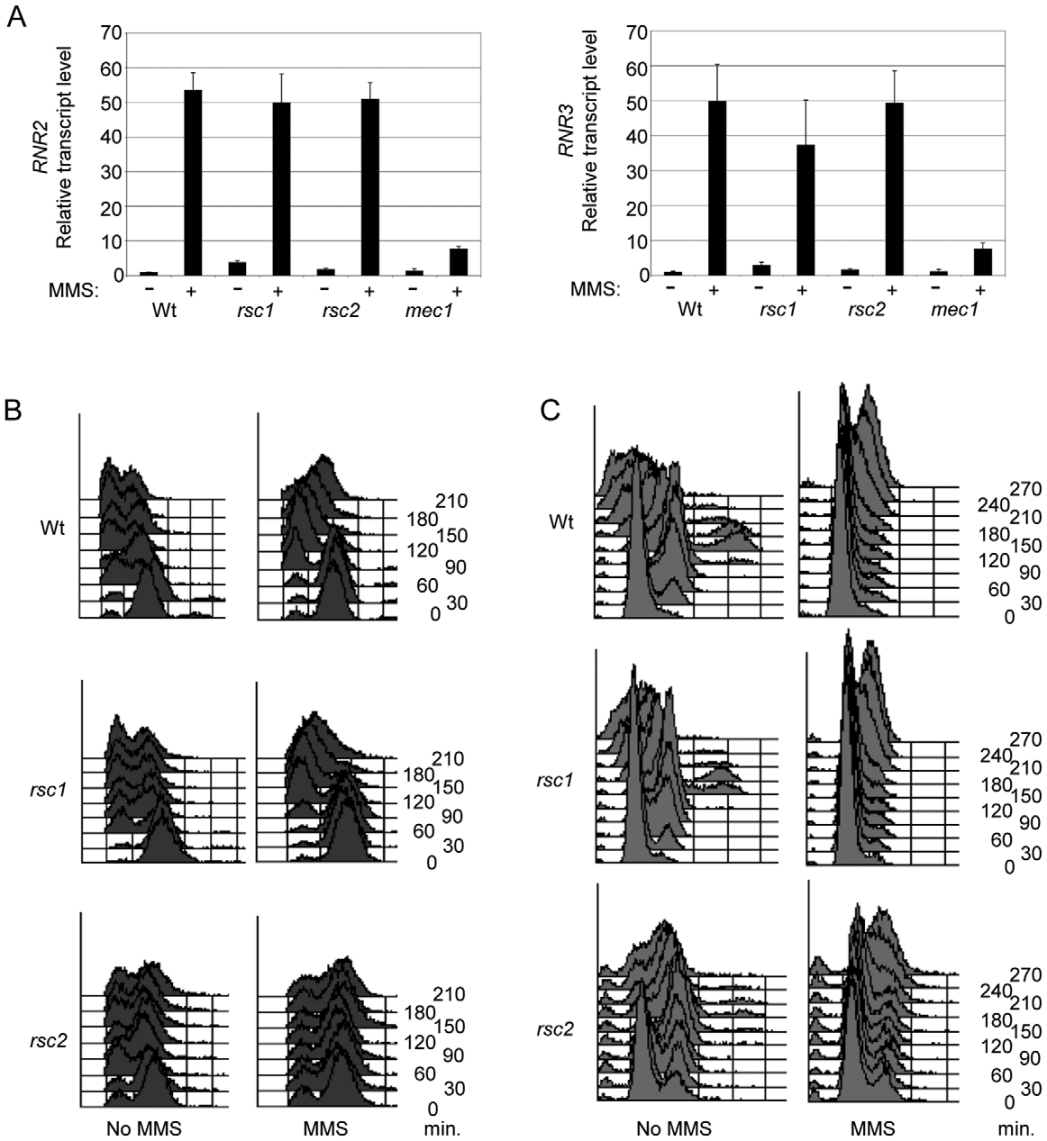
Neither Rsc1 nor Rsc2 is required for DNA damage checkpoint responses. (A) Quantitative PCR analysis of the *RNR2* (left panel) or *RNR3* (right panel) transcripts before and after 30 min treatment with MMS in the indicated strain backgrounds. (B) FACS analysis of strains arrested in G2/M and treated with no damage (left panels) or 0.1% MMS for 1 hour (right panels). Samples were collected every 30 minutes post release. (C) FACS analysis of strains arrested in G1 and treated with no damage (left hand panels) or 0.1% MMS for 1 hour. Samples were collected every 30 minutes post release.

Previously, we and others found that in the absence of Rsc1 or Rsc2, the resection of DNA ends at an HO induced DNA DSB is reduced compared to wild-type [8], [13]. Moreover, Mec1 recruitment to a DNA break and subsequent Rad53 phosphorylation was reduced, but not abrogated in the absence of RSC [13], raising the possibility that full checkpoint activation may be dependent on Rsc1 and/or Rsc2. We first tested the ability of *rsc1* and *rsc2* mutant strains to induce transcription of the *RNR2* and *RNR3* genes in response to the DNA damaging agent MMS, which is dependent on the G2/M checkpoint signaling kinases Mec1 and Rad53 [14]. Using qPCR, we quantitated *RNR* mRNA relative to a control gene (*ACT1*). As expected, the *mec1* control strain was severely defective in transcriptional induction of both *RNR2* and *RNR3* after treatment with MMS (Figure 2A). However, we found the transcript levels of both *RNR2* and *RNR3* after treatment with methane methylsulfonate (MMS) in the *rsc1* and *rsc2* mutant strains were comparable with wild-type (Figure 2A), suggesting that Mec1-dependent signalling of DNA damage is intact in these strains.

To further explore the contribution of Rsc1 and Rsc2 to DNA damage signaling, we looked at checkpoint activity by FACS analysis. The cultures were arrested in G2/M using nocodazole, one set was treated with MMS, and the cultures were released into fresh media. Under these conditions, the wild-type strain delays cell cycle re-entry by approximately 30–60 min after treatment with damage (Figure 2B). The FACS profile of the *rsc1* mutant strain closely resembled that of the wild-type strain, with a cell cycle re-entry delay in the MMS-treated sample (Figure 2B), indicating that the checkpoint activation is normal in this strain.

For reasons that are not entirely clear, the *rsc2* mutant population was unable to fully arrest following nocodazole treatment. Nevertheless, the majority of cells arrested in G2/M and, in the absence of damage, these reentered the cell cycle after the nocodazole was removed (Figure 2B). Importantly, we found that the *rsc2* mutant strain did not show premature reentry into the cell cycle following DNA damage, as one would expect for a checkpoint deficient strain. Instead, the MMS-treated cells showed a very prolonged G2/M arrest, with cells only beginning to reenter the cell cycle at the final time point of the assay (Figure 2B). This prolonged arrest may be due in part to the recently identified role of Rsc2 in promoting mitotic exit under certain conditions [5]. Nonetheless, these data indicate that the *rsc2* mutant cells are able to activate the G2/M checkpoint in response to DNA damage.

H2A phosphorylation contributes to G1 checkpoint responses and we and others found H2A phosphorylation is reduced in both *rsc1* and *rsc2* mutant cells under certain conditions [8], [11], [13]. We therefore examined the G1 checkpoint by arresting cells with alpha factor, treating one set of cultures with MMS, and releasing the cells into fresh media. Their reentry was monitored by FACS analysis, and we found that both *rsc1* and *rsc2* show a similar delay in cell cycle reentry after DNA damage as the wild-type strain (Figure 2C). Therefore, it appears that despite defects in resection and H2A phosphorylation, there is still sufficient Mec1 binding and activity to result in DNA damage induced checkpoint activation in both the *rsc1* and *rsc2* mutant strains.

### Both Rsc1 and Rsc2 facilitate NHEJ activity

**Figure 3:**
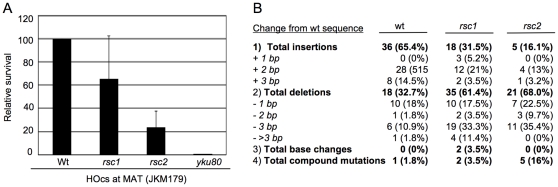
Both Rsc1 and Rsc2 contribute to wt levels of NHEJ and show a similar spectrum of repair junctions. (A) Survival of strains in the presence of continued transcriptional induction of the HO endonuclease in JKM179-derived strains (where the HOcs exists at the wild-type location at the MAT locus, but HML and HMR have been deleted). Survival is calculated as the number of colonies surviving in continued HO expression relative to conditions where HO is not expressed. (B) Summary of junctions of repair events from survivors of an HO-induced DNA DSB from wt, *rsc1* and *rsc2* mutant strains. Percent of total repair events shown in parentheses.

Because RSC is known to contribute to NHEJ [10], we decided to look at this pathway in order to further investigate the individual contributions of Rsc1 and Rsc2 to DNA repair. We tested survival after induction of the HO endonuclease, which cleaves at the mating type (MAT) locus. Normally, this break is repaired by HR using the silent mating cassettes (HML and HMR), but we disrupted *rsc1* and *rsc2* in a strain in which these cassettes have been deleted (JKM179; [15]). Survival in these strains is therefore a readout of NHEJ activity. We found that the *rsc2* mutant strains had substantially decreased levels of survival relative to wild-type (Figure 3A), whereas the survival defect in this *rsc1* mutant strain was relatively mild (Figure 3A).

In these assays, the survivors have repaired the DSB in an error-prone manner to allow growth in the continued presence of the HO endonuclease. We sequenced the junctions of surviving colonies to determine whether the spectrum of repair events is similar between the strains. We found that the *rsc* mutant strains showed a different pattern of repair junctions from wild-type (Figure 3B). Interestingly, the *rsc1* and *rsc2* mutant strains had very similar profiles (Figure 3B). Specifically, the majority of repair junctions in the wild type strains had insertions (65.4%). In contrast, both *rsc1* and *rsc2* mutant strains had fewer insertions (31.5% and 16.1%, respectively), and an increase in the number of deletions (61.4% and 68.0%, respectively, compared with 32.7% for wt). Moreover, the majority of deletion events in wt were 1 bp (56% of the total number of deletions). In contrast, there was a clear increase in the number of 3 bp (or greater) deletion events in both the *rsc1* and *rsc2* survivors.

There were only two notable differences in the repair junctions between survivors from *rsc1* and *rsc2* mutant strains. First, some of the *rsc1* mutant survivors had 1 bp insertions, but none were recovered from wt or *rsc2*. Second, we identified 5 survivors (16%) in the *rsc2* mutant strain (but not wt or *rsc1*) that had compound mutations. However, these differences are relatively subtle compared to the changes between wt and the two *rsc* mutant strains, and suggest that Rsc1 and Rsc2 may make equivalent contributions to NHEJ activity. Alternatively, Rsc1 and Rsc2 may provide distinct functions, but on the same NHEJ pathway.

### Defects due to loss of *RSC1* or *RSC2* are not rescued by additional copies of the other gene

**Figure 4:**
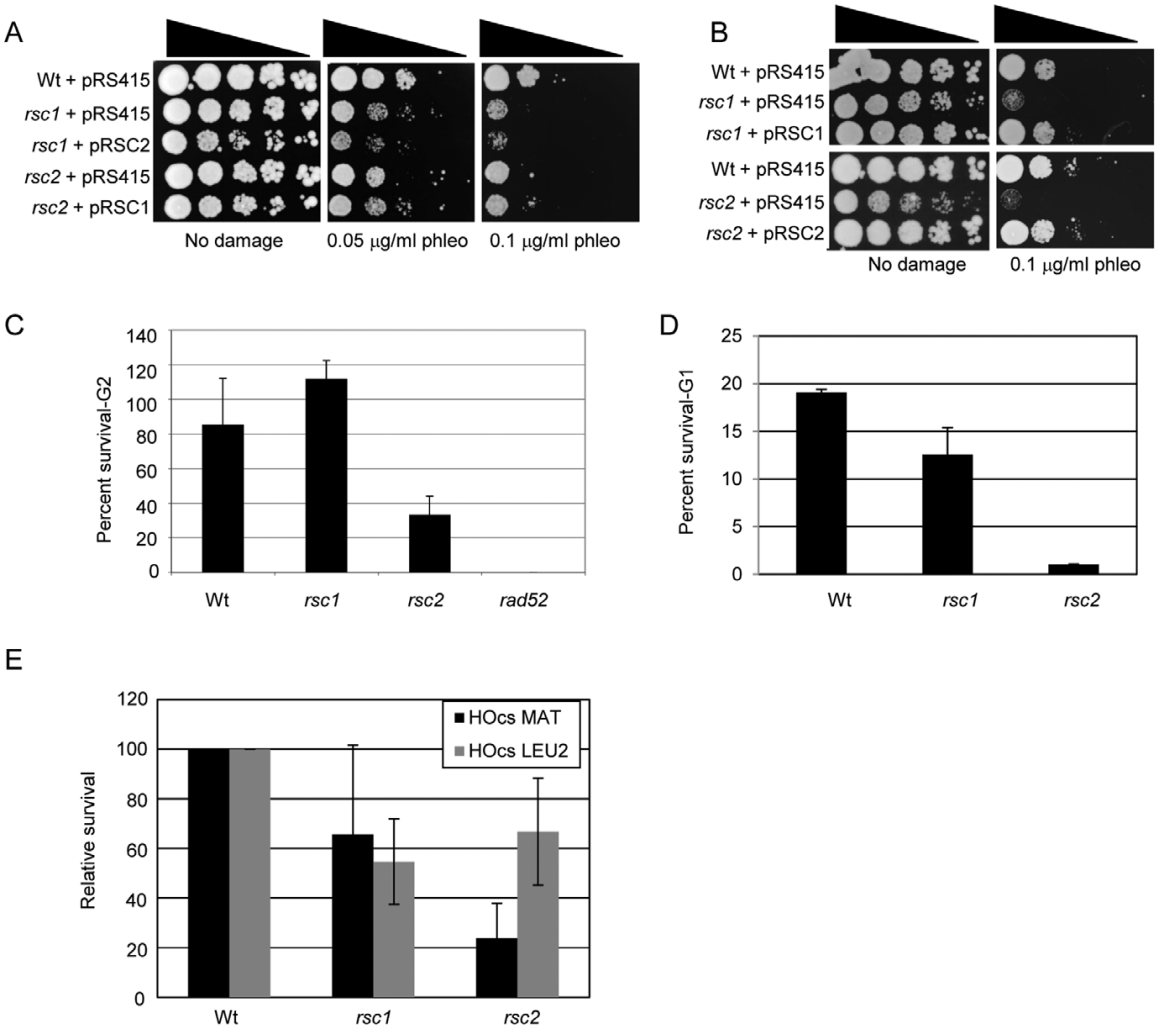
Rsc1 and Rsc2 have distinct functions in mediating DNA damage hypersensitivity. (A) and (B) Serial dilutions of *rsc1* and *rsc2* mutant strains harbouring the indicated expression construct were plated onto media containing the indicated dose of phleomycin. (C) Survival after 1 hour of 0.1% MMS treatment in G2/M arrested wt, *rsc1*, *rsc2*, or *rad52* mutant strains relative to untreated G2/M arrested cells. (D) Survival after 1 hour of 0.1% MMS treatment in G1 arrested wt, *rsc1* or *rsc2* mutant strains relative to untreated G1 arrested cells. (E) Relative survival of wt, *rsc1* or *rsc2* mutant strains in the JKM179 strain background (which carries the HO cleavage site in the MAT locus) or the YPF17 strain background (which carries the HO cleavage site in the *LEU2* gene) in the presence of continued transcriptional induction of the HO endonuclease.

To further examine the functional distinction of Rsc1 and Rsc2 in DNA damage responses, we tested the possibility that we could rescue hypersensitivity of the strains to DNA damage by increasing the dosage of the remaining gene. Therefore, we provided an additional copy of *RSC2* in the *rsc1* mutant strain and found there was no detectable change in the hypersensitivity of the strain to DNA damage caused by phleomycin or MMS (Figure 4A and data not shown). Similarly, an additional copy of *RSC1* in a *rsc2* mutant strain does not increase the DNA damage resistance of that strain (Fig. 4A). As expected, these plasmids were able to rescue DNA damage hypersensitivity when Rsc1 was expressed in a *rsc1* mutant strain and when Rsc2 was expressed in a *rsc2* mutant strain (Figure 4B). Therefore, while both proteins contribute to NHEJ and have similar phenotypes, these data suggest that Rsc1 and Rsc2 provide distinct functions in mediating survival after DNA damage and cannot entirely compensate for one another.

### Rsc1 and Rsc2 make distinct contributions to DNA repair

We noted that the *rsc2* mutant strain, but not the *rsc1* mutant strain, had a prolonged checkpoint arrest when treated with MMS in G2/M phase of the cell cycle (Figure 2B), raising the possibility that *RSC1* and *RSC2* may have different cell cycle-dependent contributions to DNA damage responses. We therefore arrested wt, *rsc1* and *rsc2* cells either in G1 or G2/M and assayed survival after exposure to 0.1% MMS for 1 hour.

We find that the *rsc2* mutant strain is hypersensitive to MMS in both phases of the cell cycle, with survival rates of 36% in G2 and 5% in G1 relative to wt (Figure 4C and D). In G1, the survival post-MMS treatment was decreased to 67% of wt levels in a *rsc1* mutant (Figure 4D). However, the *rsc1* strain did not show decreased survival relative to wt when treated with MMS in G2 (Figure 4C), suggesting that the Rsc1 isoform only contributes to DNA damage responses under these conditions in G1.

The mating type locus is a highly specialized chromatin environment that is set up to facilitate efficient gene conversion [8], [16]. We therefore investigated whether Rsc1 or Rsc2 contributes to NHEJ activity when the DSB occurs elsewhere in the genome. To do this, we deleted *RSC1* or *RSC2* in YPF17 in which the sole HO cleavage site exists in the *LEU2* open reading frame [17] and compared this with the JKM179-derived strains. When survival after HO induction was measured in this background, we again found both strains had lower levels than wild-type (Figure 4E). In this case, however, the severity of the NHEJ defect in the two strains was different to the MAT locus. The survival in the *rsc2* mutant strain was much better when the DNA DSB was induced in the *LEU2* gene than at the MAT locus (67% versus 24%; Figure 4E). In contrast, the survival level of the *rsc1* strain was not significantly different between the two strains (Figure 4E). These results suggest that the relative dependence on Rsc1 or Rsc2 depends on the context of the DSB.

### Rsc1, but not Rsc2, is required for nucleosome sliding at a DNA DSB

**Figure 5:**
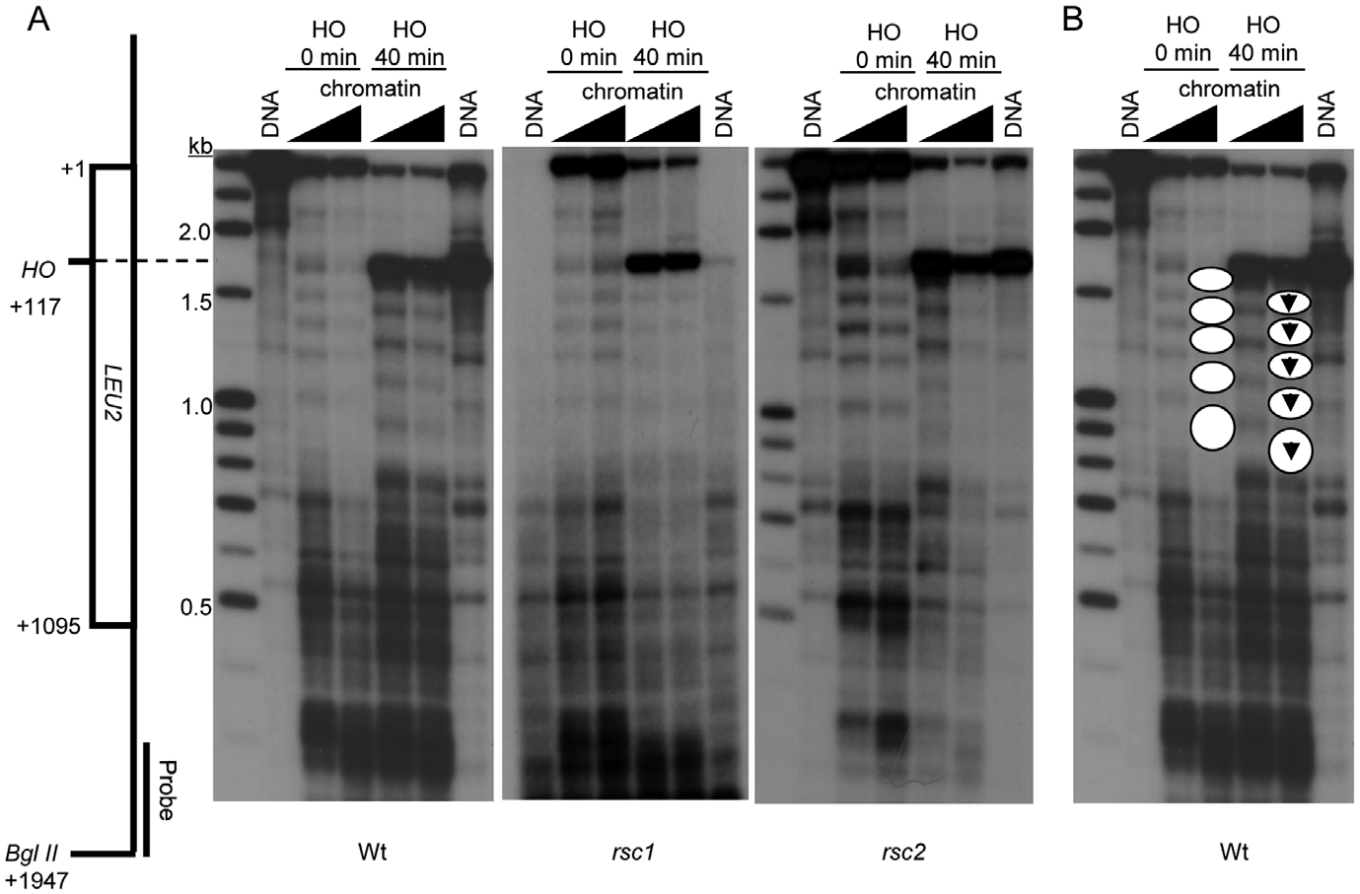
Loss of Rsc1 function, but not that of Rsc2, abolishes DSB-dependent nucleosome sliding at an HO site within the *LEU2* gene. (A) Indirect-label-analysis of MNase digested DNA from yeast strains of the YFP17 background using a probe abutting the *Bgl*II site as shown. Deproteinized DNA and chromatin samples were analyzed before (HO, 0 min) and 40 min after HO induction by addition of galactose to growth media. The HO-induced DSB appears as the strong band in HO 40 min samples at 1830 bp. (B) Inferred nucleosome positions superimposed over the wild-type MNase cleavage data from (A) to illustrate DSB-dependent nucleosome sliding associated with the HO cleavage site.

Previously, we found that the nucleosome positioning at the MAT region flanking the HO cleavage site is remodeled in a Rsc1-dependent manner [8], suggesting that the two isoforms do not provide entirely redundant functions at DNA DSBs. To determine whether this specificity also exists with a DNA DSB created in the *LEU2* gene, we tested the strains containing the HOcs in *LEU2* in our remodeling assay. First, we found that remodeling at this location results in movement of nucleosomes away from the DNA DSB (Figure 5A; nucleosome positions before and after HO induction are marked with white circles on Figure 5B). This is similar to what we found previously at the MAT locus [3]. Moreover, no nucleosomes are evicted at this time point (Figure 5). When we investigated the genetic requirements for this remodeling event, we found that remodeling at the DNA DSB in the *LEU2* gene was entirely dependent on Rsc1 (Figure 5A), similar to what we found at the MAT locus [8].

Despite the fact that both *rsc1* and *rsc2* mutant strains have reduced NHEJ activity when DNA DSBs are created at either MAT or in *LEU2*, our data demonstrate that only Rsc1 is required for the nucleosome sliding event that we detect in our assay at both genomic locations. These data further support the conclusion that Rsc1 and Rsc2 do not have entirely redundant functions in DNA damage responses.

### The BAH domain of Rsc1 is a critical determinant for the ability of RSC to remodel nucleosomes flanking a DNA break

**Figure 6:**
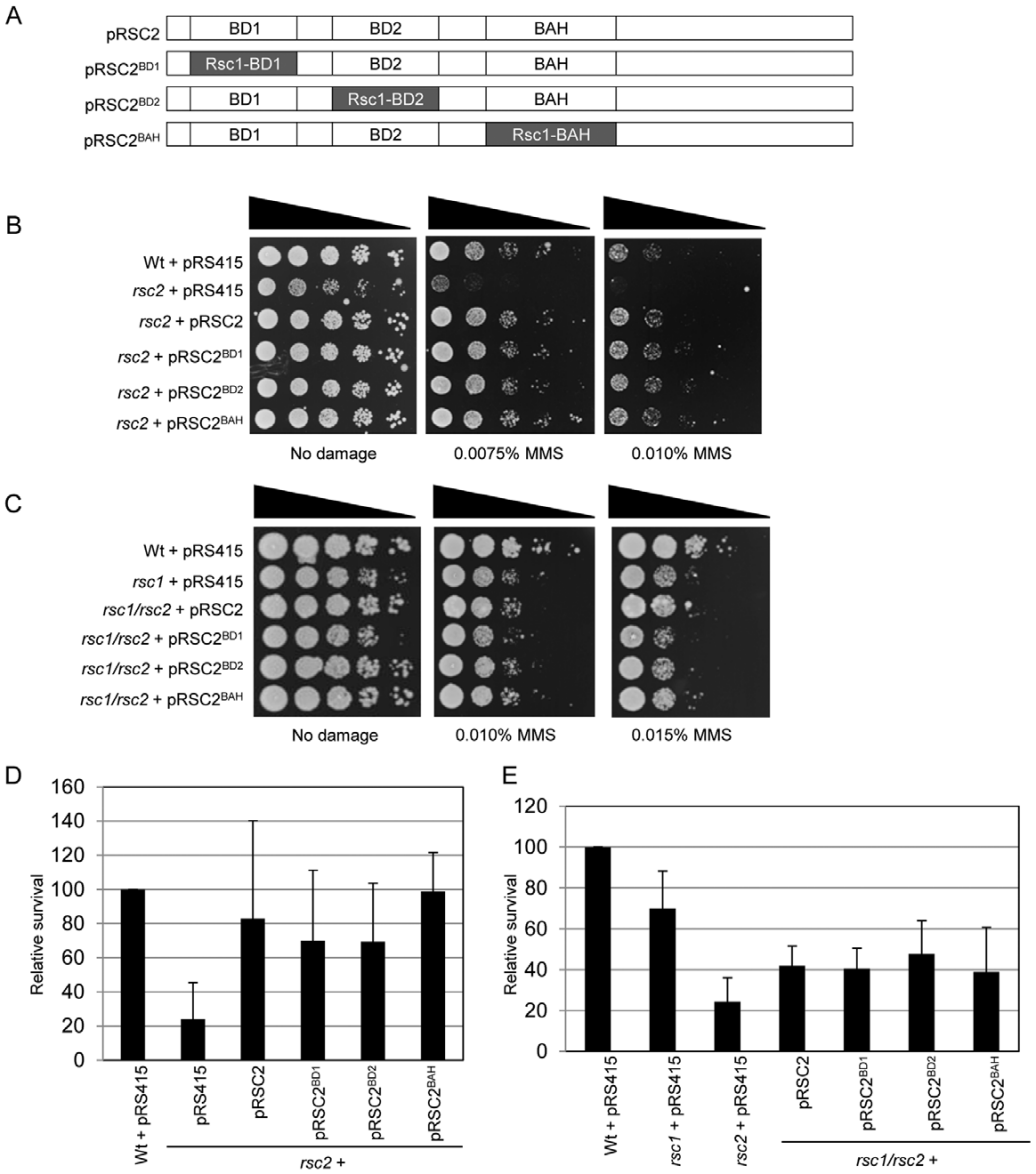
Rsc1 domains can functionally compensate for Rsc2 domains in DNA damage survival assays. (A) Cartoon of domain swapped constructs used in the assays. Bromodomain 1 (BD1), bromodomain 2 (BD2) or the bromoadjacent homology (BAH) domain of Rsc2 was replaced with the analogous domain from Rsc1. (B) Serial dilutions of mid-log cultures of wt with vector alone or *rsc2* mutant strain containing vector alone (pRS415), pRSC2, or domain swap expression plasmids were plated onto media containing the indicated dose of MMS. (C) Serial dilutions of mid-log cultures of wt with vector alone, *rsc1* with vector alone, or *rsc1*/*rsc2* mutant strain with pRSC2 or domain swap expression plasmids were plated onto media containing the indicated dose of MMS. (D) Survival of strains as in (B) in the presence of continued transcriptional induction of the HO endonuclease. Survival is calculated as the number of colonies surviving in continued HO expression relative to conditions where HO is not expressed. (E) Survival of strains as in (C) in the presence of continued transcriptional induction of the HO endonuclease. Survival is calculated as the number of colonies surviving in continued HO expression relative to conditions where HO is not expressed.

*RSC1* and *RSC2* encode similar, but not identical, proteins. Each has two bromodomains followed by a BAH domain. To investigate whether any of these three domains is critical for defining the specificity of Rsc2, we created three Rsc2 constructs in which each domain was replaced by the analogous domain from Rsc1 (Figure 6A).

First, we tested the ability of these constructs to complement a *rsc2* null strain in MMS hypersensitivity assays. Surprisingly, we found that all three constructs appeared to complement to the same degree as the wt construct (Figure 6B), suggesting that the analogous domains from Rsc1 are functional in this context. A *rsc1*/*rsc2* double mutant is inviable, so we shuffled these constructs into a strain lacking both *RSC1* and *RSC2* to determine whether they were able to support viability in the absence of *RSC1* or wt *RSC2*. All three domain swaps were viable in the *rsc1*/*rsc2* mutant background, and moreover, showed no obvious hypersensitivity to MMS compared to the wt *RSC2* construct (Figure 6C).

We then tested these constructs in the NHEJ assay as in Figure 3. We found that all three constructs rescued the NHEJ defect of the *rsc2* single mutant strain as well as the wt *RSC2* construct (Figure 6D). In addition, there was no difference in survival in this assay when the domain swaps were shuffled into a *rsc1*/*rsc2* double deletion mutant when compared with the wt *RSC2* construct (Figure 6E).

**Figure 7:**
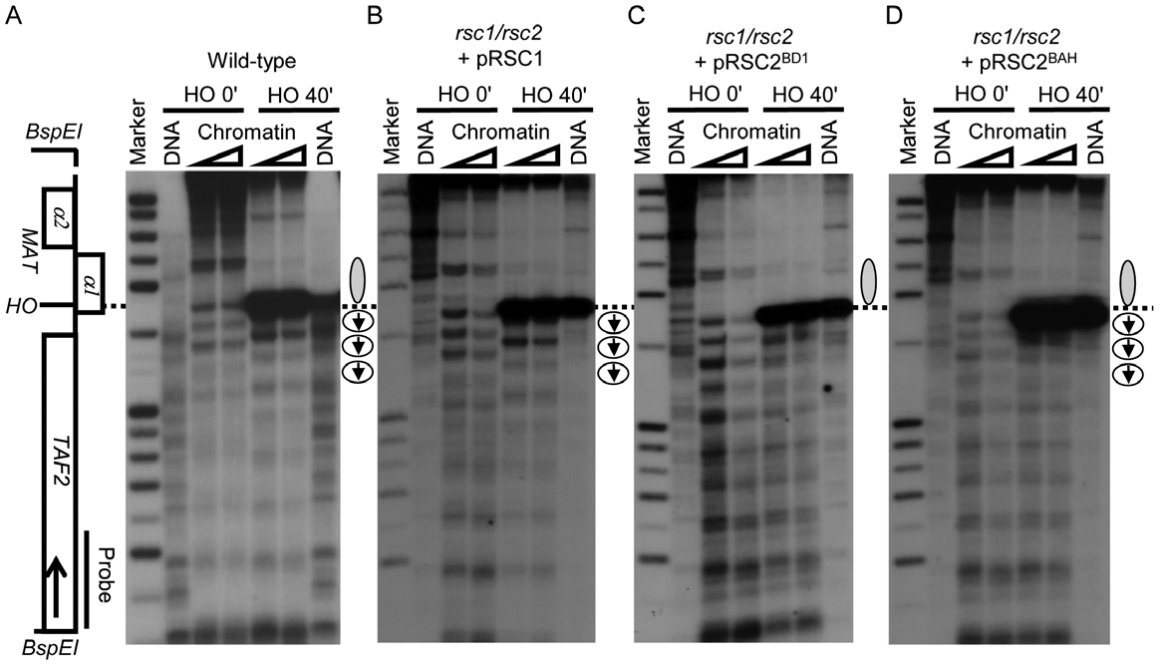
Replacing the BAH domain of Rsc2 with the Rsc1 BAH domain allows Rsc2 to remodel chromatin at DNA DSBs. (A) Indirect-end-label analysis of MNase-digested de-proteinized ‘DNA’ and ‘chromatin’ samples before (‘HO 0 min’) and 40 minutes after HO induction in *RSC1/RSC2* yeast strain JKM179 with a probe specific to the MATα locus. The position of the HO-induced DSB is marked on the gene map and across the figure with a dotted line. The nuclease-resistant structure characteristic of the normal MATα locus on the *MAT*-proximal side of the HO site is marked with a grey oval to the right of the blot. The region of DSB-dependent nucleosome sliding in the region distal to the HO site is marked with circles (representing nucleosomes) and arrows (representing the apparent direction of sliding). (B) Indirect-end-label analysis of the *rsc1/rsc2* yeast strain+p*RSC1* showing normal DSB-dependent nucleosome sliding but loss of the nuclease-resistant structure on the *MAT*-proximal side of HO (characterized by MNase cleavage sites within this region). This is consistent with the results in [3] showing that Rsc1 is required for DSB-dependent nucleosome sliding, whereas Rsc2 is required for normal MATα chromatin configuration. (C) Indirect-end-label analysis of the *rsc1/rsc2* yeast strain+pRSC2^BD1^ showing that the Rsc2-dependent nuclease-resistant structure is present as in normal cells but that DSB-dependent nucleosome sliding is defective. (D) Indirect-end-label analysis of the *rsc1/rsc2* yeast strain+p*RSC2^BAH^* showing both a normal nuclease-resistant structure together with DSB-dependent nucleosome sliding despite the absence of Rsc1.

As described above, we have a clear separation in Rsc1 and Rsc2 function in chromatin remodeling at DNA DSBs since this is entirely Rsc1-dependent. In addition, we previously showed that Rsc2, but not Rsc1, is required to establish correct chromatin structure at the MATα locus prior to DSB induction [8]. This, therefore, provided us with a very good system to investigate whether any of the individual domains influences the specificity of Rsc2. We analyzed the MATα chromatin structure before and after the induction of a DNA DSB in the *rsc1*/*rsc2* double deletion mutant harboring the domain swap constructs or the wt control. Consistent with our previous results [8], the wt strain had normal chromatin structure upstream of the HO cleavage site prior to double-strand break induction (indicated by a grey oval at the side of the MNase panel; Figure 7A), and the nucleosomes on the downstream side of the HO cleavage site are remodeled after DSB induction (indicated by the open circles with arrows; Figure 7A). Also as expected, the *rsc1/rsc2*+pRSC1 strain had altered chromatin structure upstream of the HOcs prior to induction of the DSB, but remodeling after DSB induction was normal (Figure 7B).

Strains carrying either pRSC2^BD1^ or pRSC2^BD2^ showed normal chromatin structure at the MAT locus immediately upstream of the HOcs prior to DSB induction but were unable to support substantial remodeling activity after DSB induction (Figure 7C and data not shown), similar to the pattern seen with wt Rsc2. Replacing the BAH domain of Rsc2 with that of Rsc1 also had no effect on the MATα chromatin structure, suggesting that all three domains of Rsc2 are either not required for or can support the function of Rsc2 in establishing normal chromatin structure at MATα. Strikingly, however, the strain carrying pRSC2^BAH^ was now able to carry out some remodeling of the chromatin flanking the DNA DSB (Figure 7D). This suggests that the BAH domain of Rsc1 provides some of the specificity in its role in DNA damage depending chromatin remodeling.

## Discussion

Here we show that Rsc1 and Rsc2 exist in two separate RSC complexes that appear to be otherwise identical in subunit composition. Furthermore, we find that these two isoforms of the RSC chromatin remodeling complex provide both overlapping and distinct functions in DNA damage responses, consistent with what has been found when their contributions to other cellular functions have been examined [3], [6], [7]

When *rsc1* and *rsc2* mutant strains are directly compared, the strains show very similar phenotypes - both are able to activate DNA damage signalling leading to increased RNR transcription and checkpoint activation, both show hypersensitivity to DNA damaging agents and reduced NHEJ activity, and survivors of a sustained HO-induced DSB have a very similar spectrum of repair junctions in the two strains, which are distinct from those in a wt strain. Replacing either bromodomain or the BAH domain of Rsc2 with the analogous domain from Rsc1 has no effect on the ability of Rsc2 to function in supporting survival after DNA damage, suggesting that the domains are interchangeable for at least some functions.

However, our data suggest that the two isoforms are not functionally redundant. Increasing the gene dosage of one of the two genes does not compensate for loss of the other in MMS survival or NHEJ assays. Moreover, the dependence of NHEJ activity on the presence of either *RSC1* or *RSC2* appears to be context dependent, arguing for distinct contributions. We also find that nucleosome remodeling flanking DNA DSBs at two different genomic locations is entirely dependent on Rsc1 in our assays. Interestingly, Rsc2 has also been implicated in nucleosome reorganization at chromatin flanking a DNA DSB in a study using different approaches [11]. Taken together with our finding that both Rsc1 and Rsc2 contribute to NHEJ activity, combined with the similar spectrum of repair junctions in the two mutant strains, these data suggest that Rsc1 and Rsc2 both function at a DNA DSB to mediate NHEJ via two distinct chromatin remodeling events.

Surprisingly, replacing the BAH domain of Rsc2 with that of Rsc1 results in a strain that is capable of both establishing chromatin structure at the MATα locus, which is normally Rsc2-dependent, and remodeling chromatin flanking a DNA break, which is normally Rsc1-dependent. However, while this fusion protein is capable of carrying out both Rsc1 and Rsc2-specific remodeling activities, it is still not able to compensate for loss of both proteins in survival and NHEJ assays, indicating that some functions are still absent. These functions presumably require the other domains of Rsc1 and Rsc2.

The possibility that the bromodomains and BAH domains of both Rsc1 and Rsc2 act in a combinatorial manner to mediate their functions is an attractive one since in higher eukaryotes, there are no separate Rsc1- and Rsc2-containing isoforms of the homologous chromatin remodeling complex, termed PBAF (or hSWI/SNF-B). Instead, the mammalian BAF180 (Polybromo) subunit of PBAF contains 6 bromodomains and 2 BAH domains and appears to be a fusion of budding yeast Rsc1, Rsc2 and Rsc4 subunits [18]. These domains could contribute to the functional specificity of BAF180 in a modular fashion, which may be particularly important since there are multiple alternative splice transcripts of BAF180 containing different combinations of domains [19].

To this end, we are currently investigating the structure and function of the individual bromodomains and BAH domains of Rsc1 and Rsc2 and their roles in DNA damage responses. These studies may shed light on the mechanism by which BAF180 functions as a tumour suppressor gene [20], [21].

## Materials and Methods

### Yeast strains and plasmids

Strains and plasmids used in this study are listed in Tables 1 and 2.

**Table 1:** Yeast strains used in this study.

Strain name	Genotype	Source
JKM179	*Δho Δhml*::*ADE1 Δhmr*::*ADE1 ade1-100 leu2-3,112 lys5 trp1*::*hisG ura3-52 ade3*::*GAL::HO* MATα	[15]
YNK179-177	*rsc1*::*KanMX* in JKM179	[8]
YNK179-191	*rsc2*::*KanMX* in JKM179	[8]
DDY053	*mec1*::*TRP1*, *sml1-1* in W303a	[22]
JDY1	*rad52*::*TRP1* in W303α	[23]
JDY79	*yku80*::*KanMX* in JKM179	This study
YPF17	*Δmata*::*hisG ade1 lys5 trp1*::*hisG ura3-52 leu2*::*HOcs* Δ*ho* Δ*hml*::*ADE1* Δ*hmr*::*ADE1 ade3*::*GAL*-*HO*	[17]
YNK17-20	*rsc1*::*KanMX* in YPF17	This study
YNK17-24	*rsc2*::*KanMX* in YPF17	This study
Rsc1-TAP	MATa *his3Δ1 leu2Δ0 met15Δ0 ura3Δ0* Rsc1-TAP	Open biosystems
Rsc2-TAP	MATa *his3Δ1 leu2Δ0 met15Δ0 ura3Δ0* Rsc2-TAP	Open biosystems
DMY2804	*RDN1*-*NTS1*::*mURA3* in W303a	[24]
JDY789	*rsc1*::*KanMX* in DMY2804	This study
JDY790	*rsc2*::*KanMX* in DMY2804	This study

**Table 2:** Plasmids used in this study.

Strain name	Genotype	Source
pRS415	Yeast expression vector CEN/ARS, *LEU2*	Novagen
pRSC1	Rsc1 coding sequence plus 700 bp upstream sequence and 200 bp downstream sequence in *Not*I site of pRS415	This study
pRSC2	Rsc2 coding sequence plus 700 bp upstream sequence and 200 bp downstream sequence in *Not*1 site of pRS415	This study
pRSC2-URA	Rsc2 coding sequence plus 700 bp upstream sequence and 200 bp downstream sequence in *Not*I site of pRS416	This study
pRSC2^BD1^	Rsc2 BD1 coding sequence (amino acids 18–130) replaced with Rsc1 BD1 (amino acids 10–122) in pRSC2	This study
pRSC2^BD2^	Rsc2 BD2 coding sequence (amino acids 281–377) replaced with Rsc1 BD2 (amino acids 242–337) in pRSC2	This study
pRSC2^BAH^	Rsc2 BAH coding sequence (amino acids 431–536) replaced with Rsc1 BAH (amino acids 391–496) in pRSC2	This study

### Analysis of Rsc1 and Rsc2 complex composition

Rsc1-TAP and Rsc2-TAP were purified by two-step affinity purification. 1 litre of yeast was grown to mid-late log phase, pelleted and popcorn made in liquid nitrogen. Yeast were lysed by grinding and the powder was resuspended in 4 pellet volumes of IgG binding buffer (10 mM Tris-HCl pH8, 300 mM NaCl, 0.1% NP40, 100 µg/ml PMSF, 1 µM leupeptin, 1 µg/ml pepstatin A, 0.5 µg/ml aprotinin). Lysate was centrifuged at 15,000 rpm for 10 min at 4°C before the supernatant was incubated with 200 ul of IgG Sepharose beads for 2 hours at 4°C. Beads were washed with 3×1 ml IgG binding buffer, followed by 2×1 ml TEV cleavage buffer (10 mM Tris-HCl pH8, 150 mM NaCl, 0.1% NP40, 0.5 mM EDTA, 1 mM DTT100 µg/ml PMSF, 1 µM leupeptin, 1 µg/ml pepstatin A, 0.5 µg/ml aprotinin). Beads were resuspended in 1 ml TEV cleavage buffer, 10 ul TEV protease were added to the samples, which were incubated overnight at 4°C. 3 ml CaM binding buffer (10 mM Tris-HCl pH8, 150 mM NaCl, 10 mM beta-mercaptoethanol, 1 mM magnesium acetate, 1 mM imidazole, 2 mM CaCl_2_, 0.1% NP40, 100 µg/ml PMSF, 1 µM leupeptin, 1 µg/ml pepstatin A, 0.5 µg/ml aprotinin) and 3 µl 1 M CaCl_2_ were added, and beads collected by centrifugation. The supernatant was bound to 50 µl calmodulin beads for 1 hour at 4°C, washed with 4×1 ml CaM wash buffer (CaM binding buffer with 300 mM rather than 150 mM NaCl), and eluted in 1 ml elution buffer (10 mM Tris-HCl pH8, 150 mM NaCl, 10 mM beta-mercaptoethanol, 1 mM magnesium acetate, 1 mM imidazole, 2 mM CaCl_2_, 0.1% NP40, 30 mM EGTA, 100 µg/ml PMSF, 1 µM leupeptin, 1 µg/ml pepstatin A, 0.5 µg/ml aprotinin). 20% ice-cold acetone was used to precipitate proteins and the pellet was resuspended in 20 µl 10 mM Tris-HCl pH 7.5. A mock purification was performed from lysate created from an isogenic untagged strain. Gel slices were analyzed in the University of Sussex Proteomics Centre.

### qPCR analysis of RNR induction

Cultures of JKM179, *rsc1*, *rsc2* and *mec1/sml1-1* mutant strains were grown to mid-log in YPAD. Cultures were then incubated for a further 3 hours at 30°C in the absence or presence of 0.05% MMS. RNA was reverse transcribed using Qiagen QuantiTech RT kit. For each sample, qPCR reactions were performed using primers within *RNR2*, *RNR3* and results were normalized to the *ACT1* locus. The transcript level of the normalized wild type undamaged sample is set to 1 and all other values are shown relative to this.

### G2 checkpoint analysis by FACS

Cultures of JKM179, *rsc1* and *rsc2* were grown in YPAD to mid-log and an asynchronous sample taken at the outset of the assay. Cells were arrested at G2/M by incubation with 15 µg/ml nocodozole for 1 h 45 min. Cultures were incubated for a further 45 min (with nocodozole still present) in the absence or presence of 0.1% MMS. Yeast were washed and resuspended in fresh YPAD and samples taken and fixed in ethanol at 0, 30, 60, 90, 120, 150, 180 and 210 min following release. Cells were harvested and incubated in 1 mg/ml RNaseA for 4 hours at 37°C, followed by incubation for 1 hour at 50°C in 2 mg/ml proteinase K solution. Yeast cells were pelleted and resuspended FACS buffer (200 mM Tris-HCl pH 7.5, 200 mM NaCl, 78 mM MgCl_2_). Cells were added to 50 µg/ml propidium iodide in 50 mM Tris-HCl pH 7.5, sonicated and analyzed using a FACS Calibur.

### G1 checkpoint analysis by FACS

Cultures of DMY2804, *rsc1* and *rsc2* strains were grown to mid-log in YPAD and an asynchronous sample taken at the outset of the assay. Cells were arrested in G1 by incubation with 4 µg/ml α-factor for 1 h followed by an additional 2 µg/ml for another hour. Cultures were incubated for a further 1 hr with a final 2 µg/ml dose of α-factor in the presence or absence of 0.1% MMS. Samples were taken at 0 and 30 min following addition of MMS and fixed in ethanol before washing and resuspending in fresh YPAD. Subsequent samples were taken at 60, 90, 120, 150, 180, 210, 240, and 270 min. Samples were processed and analyzed as for G2 checkpoint assay.

### Genomic NHEJ assay

Mid-log phase cultures were serially diluted and plated onto media containing either 2% glucose or 2% galactose. Colonies were counted after 3 days incubation at 30°C and repair efficiency was calculated as survival on galactose relative to survival on glucose. Repair junctions of colonies surviving on galactose were analyzed by sequencing. Each strain was assayed multiple independent times (JKM179 n = 8, YNK179-177 n = 7, YNK179-191 n = 8 JDY79 n = 3, YPF17 n = 8, YNK17-20 n = 7, YNK17-24 n = 8, and all plasmid-containing strains n = 3) and the standard deviation is shown.

### Spot tests

Yeast cultures were grown to mid-log phase, diluted to OD_600_ = 0.2, and 5-fold serial dilutions were spotted onto media containing the indicated concentration of drug. Spot tests were performed multiple independent times.

### Cell cycle survival assays

Exponentially growing cultures were arrested with either nocodazole or alpha factor and treated with 0.1% MMS for 1 hour. Following MMS treatment, cells were collected by centrifugation, resuspended in fresh YPAD and serial dilutions were plated onto YPAD. Colonies were counted following 3 days incubation at 30°C. Experiments were performed in duplicate and the standard deviation is shown.

### Indirect-end-label analysis

Chromatin digestion using micrococcal nuclease (MNase) was performed exactly as described [8]. DNAs were digested to completion with *Bgl*II or *Bsp*EI (for analysis of the LEU2 and MAT loci, respectively) and separated on 1.5% agarose gels. Probes were 400 bp fragments abutting the appropriate restriction site and prepared by PCR.
